# Comparing the effectiveness of hyperspectral imaging and Raman spectroscopy: a case study on Armenian manuscripts

**DOI:** 10.1186/s40494-018-0206-1

**Published:** 2018-07-06

**Authors:** Ian J. Maybury, David Howell, Melissa Terras, Heather Viles

**Affiliations:** 1 School of Geography and the Environment, Oxford University Centre for the Environment, University of Oxford, South Parks Road, Oxford, OX1 3QY UK; 2 Weston Library, Broad Street, Oxford, OX1 3BG UK; 3 College of Arts, Humanities and Social Sciences, University of Edinburgh, Edinburgh, UK

**Keywords:** Hyperspectral imaging, Raman spectroscopy, Pigment identification, Illuminated Armenian manuscripts, Manuscript studies, Multispectral imaging, Special collections

## Abstract

There is great practical and scholarly interest in the identification of pigments in works of art. This paper compares the effectiveness of the widely used Raman Spectroscopy (RS), with hyperspectral imaging (HSI), a reflectance imaging technique, to evaluate the reliability of HSI for the identification of pigments in historic works of art and to ascertain if there are any benefits from using HSI or a combination of both. We undertook a case study based on six Armenian illuminated manuscripts (eleventh–eighteenth centuries CE) in the Bodleian Library, University of Oxford. RS, and HSI (380–1000 nm) were both used to analyse the same 10 folios, with the data then used to test the accuracy and efficiency of HSI against the known results from RS using reflectance spectra reference databases compiled by us for the project. HSI over the wavelength range 380–1000 nm agreed with RS at best 93% of the time, and performance was enhanced using the SFF algorithm and by using a database with many similarities to the articles under analysis. HSI is significantly quicker at scanning large areas, and can be used alongside RS to identify and map large areas of pigment more efficiently than RS alone. HSI therefore has potential for improving the speed of pigment identification across manuscript folios and artwork but must be used in conjunction with a technique such as RS.

**Electronic supplementary material:**

The online version of this article (10.1186/s40494-018-0206-1) contains supplementary material, which is available to authorized users.

## Introduction

### Pigment analysis

Technical investigations of works of art are of great value to conservators and researchers. Scientific techniques have been applied to pigment identification as part of conservation since the late twentieth century [1, 2] aiding: characterisation of the palette of an artist or workshop [3, 4]; art historical understanding of the artist [5]; identification of restorations or interventions [3, 6]; monitoring degradation [7]; detailing the conservation state of an item [3]; revealing preparatory sketches [6], underdrawings [3], or palimpsests [8]; segmentation of a painting into regions for differential processing such as colour retouching [9]. Such knowledge is of value to researchers and can also aid in effective conservation strategies and restoration [7], and answer questions of provenance [3, 4, 6, 7, 10–12]. In the broader sense it can also help determine trade routes and cultural interactions [12, 13], and give some idea of the technology of the period with respect to pigment manufacture [12, 13].

Sampling-based techniques such as gas-chromatography–mass spectrometry (GC–MS) and high performance liquid chromatography (HPLC) provide considerable data [14] however a combination of non-destructive techniques generates desirable data without harming the item (an obvious advantage for conservators) [14, 15]. A combination of techniques is most often used, as any one technique has limitations. For example, Mosca et al. [16] used Raman spectroscopy and X-ray fluorescence spectroscopy (XRF) to identify pigments in different layers of illuminated manuscripts. Other techniques include: fibre optic reflectance spectroscopy (FORS) [14], and Fourier transform infra-red spectroscopy (FT-IR) [17].

Access to these techniques and approaches for cultural heritage imaging can be restricted due to their cost, availability, and the complex nature of interdisciplinary research [18]. It is therefore useful to assess which approaches are most feasible, cost-effective, efficient, and accurate for our purpose of pigment identification, as this will help scholars understand the affordances of different systems and aid in the scoping and management of programmes of research.

### Raman spectroscopy

Raman spectroscopy is a common and relatively accessible method in pigment identification [4, 19–23], it can be used as a benchmark when assessing the application of HSI to this problem.

Raman spectroscopy has been used to look at significant works of art by artists such as Picasso [21, 24] and Vermeer [10], and also at high value items such as the Lindesfarne Gospels [25] and high profile forgeries [5, 26]. Raman has proved to be efficient at pigment identification [5, 7], although there are some exceptions such as lakes^1^ and other organic pigments which have poor Raman scattering, making identification difficult [14]. Where Raman scattering is poor, useful results have been obtained by combining with XRF [3]. RS has also been used to investigate the distribution of pigments and extent of restoration by looking at cross sections of objects [21].

Since its introduction as an analytical technique for the study of heritage in the 1970s, RS has become smaller, portable, and simple enough to be used by those who are not considered experts or analysts [19, 27].

Although accurate, it is also very time consuming to use Raman spectroscopy to identify pigments across large areas, as sample points are small and have to be repeated. Raman mapping has been used to examine areas on the micrometre scale over several hours. For example, one archaeological study examining rust took 21 h to image a 52.2 × 46.2 µm rectangle [28], another looking at cross sections of a sixteenth century painting took 4.5 h to image a 60 × 45 µm rectangle [29]. An area can be mapped simultaneously by defocussing the laser beam, but this greatly reduces the intensity and as such is limited to a field of view on the order of micrometres [30]. In general using Raman spectroscopy as a mapping tool is unusual [31]. XRF has also been used for mapping but is also very slow, with an area of 36 × 34 mm scanned in 45 min in one study [16]. In contrast, hyperspectral imaging can image an A4 sheet (210 × 297 mm) in roughly 15 min. It is therefore worth considering if other techniques such as HSI can be used in conjunction with RS to increase efficiency in identifying pigments, particularly across larger areas.

### Hyperspectral imaging

Hyperspectral imaging (HSI) is a reflectance technique which, for each pixel in an image, produces a reflectance spectrum for the wavelength range detected [1]. This holds many potential advantages for pigment identification on a large scale given that it scans large areas quickly, and it is this property (or affordance) that we wish to investigate, within the context of pigment identification. The spectra produced can be used to characterise the materials at the surface of the image and produce maps of these materials, but hyperspectral imaging has also been used to reveal hidden images and text, given its ability to detect reflectance at many wavelengths, often including wavelengths outside of the visible range [1, 32]. Originally a remote sensing technique [32] it has been developed over the years for astrophysics, military applications, medical imaging, and more recently for the non-destructive investigation of works of cultural heritage [6, 33–35]. HSI has been successfully applied to a range of heritage material including The Declaration of Independence [36] where alterations to text were revealed, and Edvard Munch’s “The Scream” where the pigments used were characterised and mapped [37].

Multispectral imaging (MSI) and HSI have both been used as the first step in investigations to provide spatial and spectral data on the pigments in a manuscript. This then allows one to examine and map the entire surface of an object [6, 34, 38] and guides the use of techniques such as Raman spectroscopy [39, 40], XRF [41, 42], and FORS [43] which give a more detailed analysis [14, 44–46]. This methodology can overcome concerns that point-based analyses may not represent the whole and may not be sufficient to demonstrate the diversity of colourants in such works [3]. Working with a combination of techniques allows for the best results to be generated [47, 48] and it is mainly recommended to use HSI as the first technique in combination with others [6, 14].

The identification of materials in works of art via HSI can be done by comparing the reflectance spectra for the relevant pixel to those of a reference database or by the creation of false colour images from the hyperspectral data as per Haymen-Ghez et al. [49]. Such false colour composite images have recently been successfully used to aid the identification of watercolour pigments in eighteenth century botanical illustrations by Ferdinand Bauer [50], this study however focusses on the former identification method.^2^ There is no such database suitable for the identification of pigments and so one must be manufactured by the analyst for each new study. Ideally this would contain all possible combinations of colourants, binders, etc. likely to be found in the studied object and would also be historically accurate (e.g. using the same substrate as found in historic artworks). Such a level of detail is required because all of the chemical components within the area observed by a pixel contribute to the reflectance spectra and is therefore not molecularly specific and in mixtures one peak may obscure the characteristic peak of another compound. Despite this the differentiation of two colourants which are similar visually but chemically distinct (metamers) by HSI has been demonstrated [14, 21, 47, 51–53].

The question of whether or not the algorithms being used in the industry at present to analyse hyperspectral datacubes give the best results has been raised, and newer algorithms have been suggested which can take full advantage of the increasing information [33]. Doubts about the ability of HSI to identify a pigment definitively have been raised due to the complications of mixtures or degraded pigments [1]. For example, for the identification of mixtures, linear spectral unmixing was designed for remote sensing (the collection of surface data from afar, for example the geological survey of several kilometres of land using sensors on an aeroplane) where the signal is a combination of the spectral responses of spatially separated materials [1]. In such a study a pixel may be several metres across and cover both a patch of grass and some tarmac, whereas for paint materials this is not the case, as pigments are uniformly dispersed in the binding media and the spectral response is not a simple linear mixture of the reflectance. The Kubelka–Munk (KM) theory is more appropriate [1, 9] and is used in the paint industry to calculate the ratios of paints to match a given colour [1]. The effect of binding medium, particle size, and concentration have been systematically studied and dirt and varnish can also have an effect [1].

Reflectance spectra of pigments in the UV VIS range have relatively few broad bands for use in fingerprinting (compared to IR and RS) and cannot therefore always provide unambiguous identification especially when two or more absorbing species are present in a mixture. It is interesting to note that the HSI equipment used in this study has a spectral resolution of 0.67 nm which is more than sufficient [54, 55] to discern such peaks and troughs as may be present in the reflectance spectra involved and may even lead to data redundancy [55]. This would slow down any computational work done on the reflectance spectra (as the computer would carry out calculations on all the data regardless of its redundancy). Care must also be taken to ensure that relevant analysis is done, techniques used for multispectral image analysis may not be appropriate.

Identification can therefore rely on the fact that relatively few colourants could be used in any one piece due to limitations of geography, availability, and time period [14]. Unusual or unexpected colourants can create a need for the use of multi-technique analyses [14]. On the other hand, HSI has been shown to successfully differentiate between colourants that other techniques find difficult [56]. Red lake pigments (madder, for example) are very light sensitive and so their characterisation and any data on their degradation can be of upmost help [56]. HSI was used to discriminate between madder, cochineal, and brazilwood however the addition of binding media etc. made it more ambiguous. A more comprehensive database was recommended [56].

This study therefore investigates how well HSI can be applied to pigment identification given the questions raised about the appropriateness of algorithms designed for other uses, and databases which are unlikely to be complete.

## Materials and methods

### Armenian manuscripts

Hyperspectral imaging was used here to identify the pigments in a set of Armenian illuminated manuscripts from the Bodleian Library, University of Oxford, and the identifications made were compared to data obtained from Raman spectroscopy analysis of the same texts. These texts were part of an exhibition entitled “Armenia: Tales From an Enduring Culture” [57] which marked the centenary of the Armenian genocide during WW1 and displayed manuscripts felt to be representative of Armenian culture [57]. The texts were surveyed prior to exhibition and it was desired to learn about the palette used in their illumination. As a result of analysis prior to this study the exhibition was able to display the raw ingredients for the palette alongside the manuscripts in the exhibition hall.

**Fig. 1:**
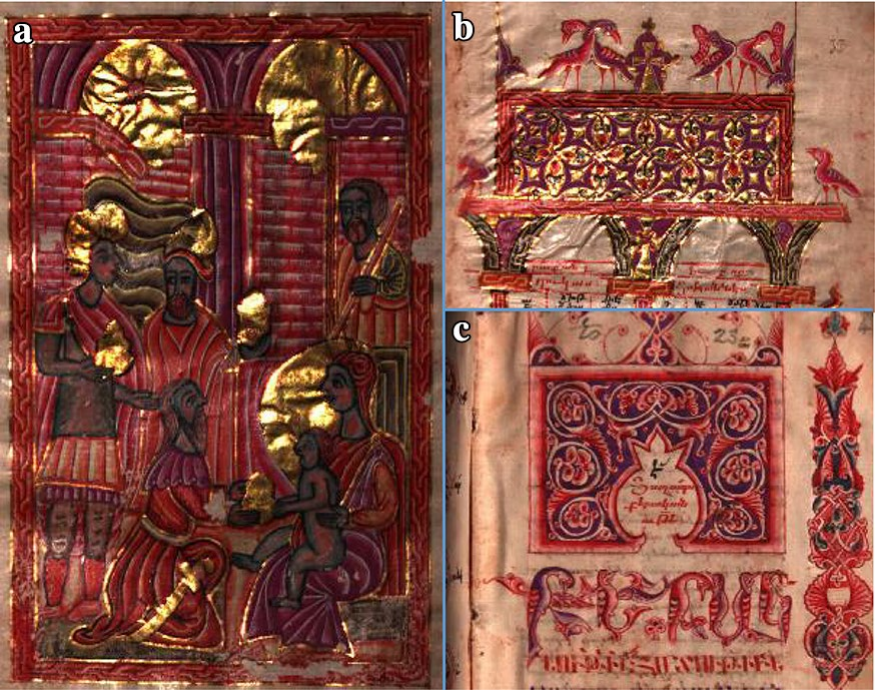
Armenian Manuscripts held by the Bodleian Library. **a** MS Arm. d13 (1609 CE) folio 4r: The adoration of the Magi, **b** MS Arm. d13 folio 33r: Eusebian canon table. This manuscript was the work of Mesrop, a famous Armenian illuminator, **c** MS Arm. e34 folio 4r: The first page of the Armenian translation of the classical Greek grammar attributed to Dionysius Thrax “Concerning Grammar”. Dated eighteenth Century

Illuminated manuscripts of Armenia, while visually stunning, have not been the subject of many scientific studies [58, 59] yet they are historically varied and of much scholarly interest [60–65]. The Bodleian Library has over 140 manuscripts, and over 250 early printed books in its Armenian collection with a date range of over 1000 years acquired from 1635 onwards [66]. A few examples of these, used during this study and displayed during the exhibition, are shown below in Fig. 1.

Armenian illuminators have left no written account of how they prepared their pigments [60]. One study begun in 1979 identified six pigments as the staple Armenian palette with differences in recipes and quality accounting for differences in locale and era. They are gold, white lead, vermillion, orpiment, ultramarine, and red lake.^3^ Mixtures of these six pigments are commonly used to create different hues and shades [61, 67]. It should be noted the study was of 24 manuscripts, which is a small percentage of the total known today. Two things may strike the art historian as odd about the palette: firstly, the use of ultramarine as the standard pigment [68]. In European art, ultramarine is rare [68]. It is made from lapis lazuli mined in present day Afghanistan, making it more expensive for European artists than for Armenian artists where it is thought that the close proximity to the mines meant that costs were kept low [68]. Secondly, the lack of a natural green pigment (organic or inorganic) such as verdigris, which meant the artists had to use a mixture of other pigments to achieve the green colour instead [60, 68]. Mixtures of mineral yellow, orpiment, and blue (either ultramarine or an organic blue) with lead white to alter the shades were used to produce an olive green which was duller than that found in western manuscripts [59, 67–69].

The Bodleian Library’s collection, spanning all of the aforementioned periods of production makes it ideal for carrying out a study of the palette used. Working with a curator, a range of six manuscripts dated between the fourteenth and eighteenth centuries, including works by well-known artists such as Mezrop (MS. Arm. d.13, Gospels, 1609) were chosen for this study.

The corpus studied is replicated in Table 1 below, and ranges from the eleventh to the eighteenth century.

**Table 1:** Details of the Armenian manuscripts from the Bodleian Libraries selected for this study

Shelf mark	Page	Date	Title
MS. Arm. e.34	4r	Eighteenth century	Grammatical and philosophical tracts eighteenth century
MS. Arm. d.13	22r	1609	Gospels
MS. Arm. d.3	10r	1304	Gospels
MS. Arm. g.4	N/a	1706–1707	Phylactery
MS. Arm. d.22	8r	Late sixteenth century	Gospels
MS. Arm. c. 3	1r	Sixteenth century	Menologium sixteenth century

### Workflow

To evaluate the utility of HSI with respect to the identification of pigments using the software ENVI^4^ and the characterisation algorithms contained within in comparison to RS it was necessary first to gather the point-based RS data. HSI could then be carried out on the folios from which RS data had been gathered. It was then necessary either to compile a database of reflectance spectra or utilise an available one. This database could then be used for the identification of the HSI reflectance spectra by way of a comparison between the data gathered and the known spectra in the database. This comparison is done by way of three computer algorithms available in the ENVI software [spectral angle mapping (SAM), spectral feature fitting (SFF), and binary encoding (BE)^5^], which are to be compared to each other in order to suggest which one is the most applicable to this data. The HSI data could then finally be compared to the RS data (which is identified separately by way of characterising peaks in the RS spectra).

**Fig. 2:**
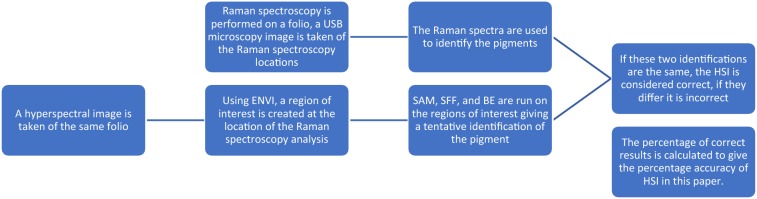
A flow diagram of the experiment, explaining the origin of the percentage accuracy for HSI

Pigments in the manuscripts were identified based on their spectral signature from Raman spectroscopy. 90 sample spots were analysed from 10 folios from the six manuscripts (one would normally analyse fewer spots to characterise the pigments in the manuscripts but more were desired to give this study sufficient data). Setting up Raman spectroscopy takes about 10 min and a further 5–10 min for analysis of each subsequent spot. Not all pigments provide strong Raman scattering signals and so this study focused on those which gave good signals using the Raman spectroscopy equipment available (see below), in order for the HSI setup to be compared to a technique known to be accurate. The six pigments the study focussed on were vermillion, indigo, lapis lazuli, red lead, red lead/vermillion mixture, and indigo/orpiment mixture. Gilded regions of the manuscripts generated unwanted specular reflectance and so analysis was done on regions unaffected by this phenomenon. HSI was then performed on the same manuscripts and used for the identification of the same pigments as the Raman spectroscopy equipment. The two results were then compared directly and if a pigment was characterised as the same pigment by HSI as it was Raman spectroscopy it was considered correct. The percentage of the total characterised which were correct (i.e. generated the same result as from Raman spectroscopy) was considered to be the percentage accuracy. In order to classify reflectance spectra a comparison to a reference database is carried out, as explained below. Raman spectroscopy did not require the creation of a database from scratch. The workflow is represented diagrammatically below in Fig. 2.^6^

### HSI database

**Fig. 3:**
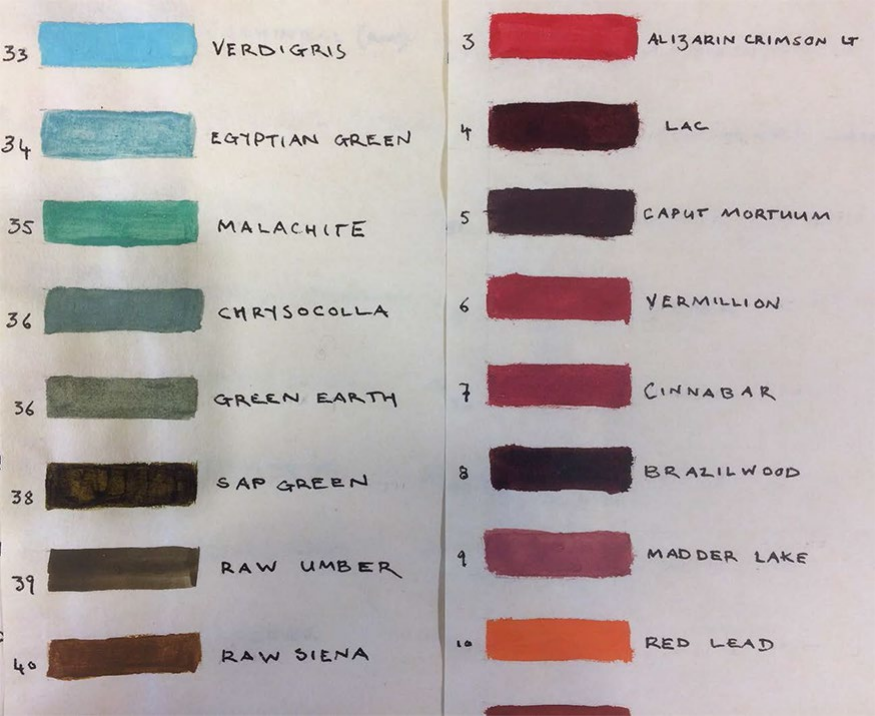
Colour swatches of different pigments including common mixtures. These were used to make database 1 (and part of 3)

Reference libraries were needed for the characterisation of the reflectance spectra obtained from hyperspectral imaging. An initial reference library for reflectance spectra was therefore made, using 113 colour swatches painted using Kremer paints^7^ (a sample of which are shown below in Fig. 3) on a paper thought to be similar to an eighteenth-century artist’s in order to replicate as closely as possible the spectra expected from the genuine manuscripts.

The first database was created from these colour swatches taking the most likely pigments to be found in Armenian texts based on studies of Armenian illuminated manuscripts [60, 68] and eliminating the ones which one would not expect to find (synthetic pigments for example). This first database was later improved upon once some HSI scans had been taken of some of the Armenian manuscripts and used to create another, more up to date database which included the Armenian spectra, and a further database created which had exclusively the Armenian spectra in it. These three databases were compared with each other in order to see the effect of a change in database on the identification accuracy. Two further databases were also used. A database was downloaded online [70, 71] to form database 4, and of these the more likely pigments for Armenian illuminated manuscripts were taken to form database 5. These were used so that externally made references could be compared to those created for the paper. These databases had greater spectral resolution (0.19 nm) and were taken using FORS equipment which measured from 360 to 1000 nm (Table 2).^8^

**Table 2:** Showing which spectra are in which database and how many spectra each contains

Database	1	2	3	4	5
Spectra from Kremer paints	40	0	40	0	0
Spectra from Armenian manuscripts	0	18	18	0	0
Spectra from online database	0	0	0	55	11
Total number of spectra	40	18	58	55	11

Spectra from Kremer paints were of the following pigments: Smalt, gamboge, azurite, verdigris, yellow lake, realgar, massicot, orpiment, alizarin crimson, cochineal, lac, vermillion, madder, red lead, Chilean lapis, indigo, verdigris + indigo, lac + vermillion, red lead + vermillion, realgar + indigo, vermillion + cochineal, lead white.

Spectra taken from the manuscripts and used for database 2 (and later 3) were: indigo, indigo + orpiment, red lead, lapis lazuli, vermillion, red lead + vermillion.

Database 4 contained burnt umber, raw umber, vandyke brown, burnt sienna, raw sienna, red ochre, red lead, cadmium red, alizarin, madder lake, lac dye, camine lake, vermillion, realgar, yellow lake, massicot, yellow ochre, gamboge, naples yellow, lead tin yellow (two variations), saffron, orpiment, cobalt yellow, cadmium yellow, chrome green, cobalt green, cadmium green, green earth, viridian, phthalogreen, verdigris, malachite, blue bice, cobalt blue, azurite, Egyptian blue, ultramarine, phthaloblue, smalt, indigo, mayablue, Prussian blue, cobalt violet, ivory black, vineblack, boneblack, lamp black, gypsum, chalk, leadwhite, zinc white, titanium white, lithopone, cardboard.

Database 5 contained red lead, madder lake, vermillion, realgar, massicot, orpiment, Verdigris, azurite, ultramarine, smalt, indigo.

The databases were made within the ENVI software by selecting pixels from the area of the pigment, averaging the spectra, and adding this averaged spectrum to an ENVI database file. Databases 4 and 5 were simply downloaded and saved in ENVI database file format.

### Equipment

#### Hyperspectral imaging

The hyperspectral camera used was a Headwall Photonics VNIR 1003B—10,147 which detects 972 contiguous wavelengths from 380 to 1000 nm and has a spectral resolution of 0.64 nm. The lens used is a Schneider XNP 1.4/17—0303 with a headwall ACOBL—380—49X, 075 filter.

The setup is a line scanning method, and there are 1600 pixels per line scanned. The spatial resolution depends on the distance between the detector and the subject which can be adjusted at the operator’s discretion. A typical pixel size during this study was 110 µm across (this is the “ground sampling distance” the detector’s pixel size is 0.65 nm).

The light source is a halogen bulb controlled by a Techniquip 21DC, and cooled by a Minebea Motor Manufacturing Corporation 3110KL—04 W—B50 fan.

The detector remains stationary while the subject is placed on a stage which moves along only one axis, thus scanning the object. The speed of the stage is calculated based on the distance between the stage and the detector in order to ensure firstly that the pixels produced represent square sections of the object (i.e. there is no stretching or squashing effect produced) and secondly to ensure that no part of the object is missed by the scan, given that at set intervals a snapshot is taken in order to build up the hyperspectral data cube. The stage uses a Vexta PK264A2A—SG3.6 stepping motor controlled by a Velmex VXM stepping motor controller. All aspects of the setup were supplied by Headwall Photonics, including the light source and stepping motor.

As HSI gives values of reflectance, calibration is performed by giving the software an example of 100% reflectance, and 0% reflectance, these are the white and dark calibrations respectively. White calibration is carried out using a piece of Spectralon^®^,^9^ and dark calibration is performed by covering the lens with its lens cap. The room lights are off during calibration and scanning as the fluorescent lights produce unwanted lines in the scan, ideally the room would be in darkness so that any fluctuations in light intensity and thus reflectance values are eliminated. Equipment calibration (such as signal linearity and accuracy of the wavelength axis) are performed by Headwall prior to shipping.

**Fig. 4:**
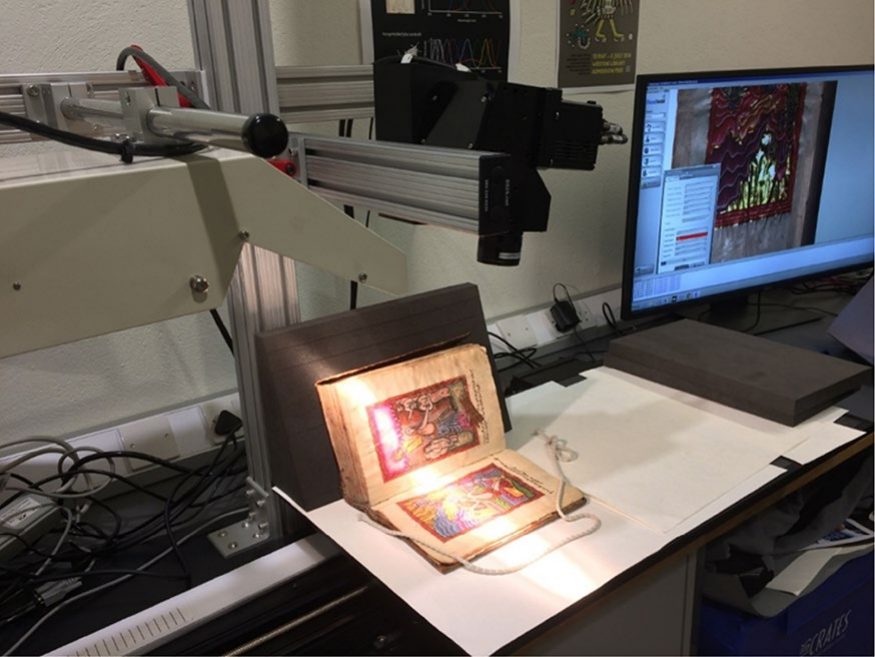
The HSI equipment during a scan of MS Arm. d13. For the purpose taking this photo of the equipment the room lights have been left on, during a scan the room lights are always off

This experimental setup is shown below in Fig. 4. During the scan the manuscript was supported when needed by manuscript grade archival book supports.

#### Data processing

For processing the Hyperspectral data cube (post-acquisition) the software ENVI was used (version 5.3.1). The spot scanned by RS had been recorded by taking a picture using a USB microscope (Veho Discovery VMS-004 delux, 400×, 2 megapixels magnification) and using the ENVI region of interest tool an average spectrum was calculated from the pixels in the same area of the RS measurement. The RS spot size was 30 µm and the pixels of the HSI were ca. 78–130 µm wide (mean pixel size: 110 µm). The HSI pixel data was analysed using the spectral analyst feature which compared, using SAM, SFF, and BE, the average spectrum for each region of interest with the spectra held in the HSI reference libraries made by us (see “Equipment” section below). Each computational method produced is a ranking where the highest score represents the closest match to the reference spectra (example given in Additional file 1). All rankings are between 0 and 1, the higher the score the closer the match for the spectra. The algorithms match the spectra in the database to the spectrum obtained during experimentation. Thus, if a relevant, trustworthy database is used a likely candidate for the identity of the pigment is given.

Using the software it was also possible to select the wavelength range over which the calculations (SAM, SFF, and BE) were performed and this was done in order to remove from the calculation either noise, or large components which were common to all scans, thus improving the agreement between the hyperspectral data and the Raman spectroscopy data. For example, some fluorescence was seen between 380 and 400 nm and spectra tended to level out after 800 nm. These parts of the spectra also coincided with the noisy regions and so the wavelength range upon which the calculations were performed was reduced in order to focus on the areas which gave identifiable peaks or troughs in the data and eliminate areas which were similar in all spectra, thus aiding the computational differentiation process (‘tailored wavelength range’ in Table 4). This reduction was made on a case by case basis in order to account for any variation in major peak positions between the different pigments, however it was found that this took a very long time as it had to be done manually. By way of comparison the computations were run again using the region between 400 and 800 nm each time to see if results could be improved upon without having to tailor the wavelength range to each individual spectrum (‘400–800 nm’ in Table 4).

The techniques are included in the software as standard as they have been used for the analysis of remote sensing data (SFF [73–75], SAM [76–78], BE [79–81]). For the analysis of heritage based hyperspectral data only SAM appears to have been used previously [42, 82, 83].

#### Raman spectroscopy

**Fig. 5:**
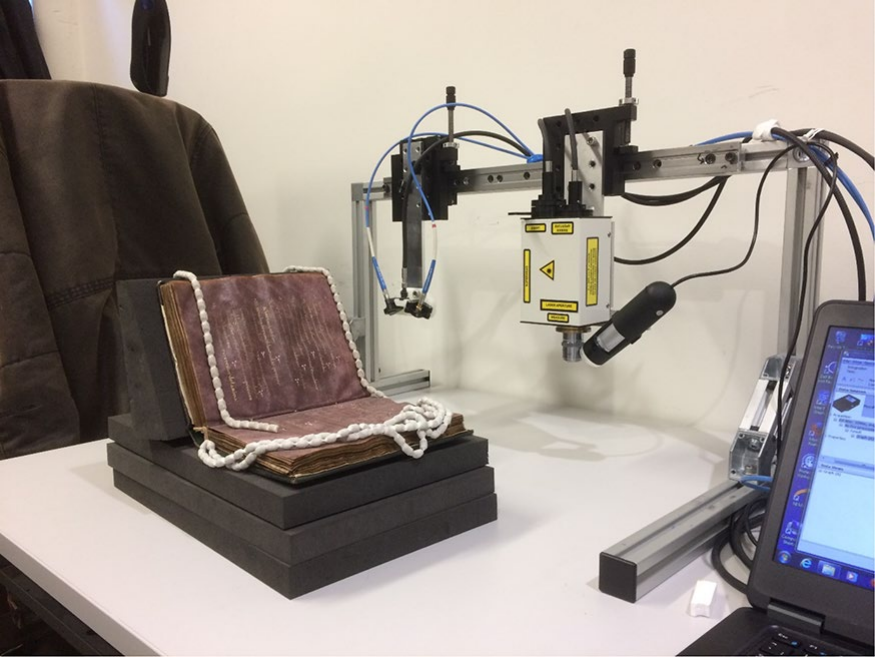
The Raman spectroscopy equipment used during the project

The Raman spectroscopy equipment used in this study has been developed specifically for the identification of pigments in objects of cultural heritage, and is unique [84]. It differs from commercial equipment primarily in that large manuscripts can fit under the detector in situ and it is extremely portable. The device shown in Fig. 5 demonstrates the sensor is attached to a lightweight frame which allows the sensor to move along in one direction with movement in the other direction being achieved through repositioning of the frame. As per HSI the manuscript is supported, if needed, by archival grade foam book supports.

Light and dark calibration are performed and a reference sample is used to correct for any shift from true peak values. All Raman measurements require a blacked-out room to minimise interference for this very sensitive technique.

The spot size of each measurement is ca. 30 µm and a JDSU 1 mW HeNe laser source is used with a wavelength of 632.8 nm. The detector measures in the range of 2577.367–91.14842 cm^−1^ and is a Andor Shamrock 163/iDus 416 camera CCD spectrograph with a Horiba Ltd Superhead sampling accessory. A USB microscope generates a live image on the laptop, stills of which are used to record the exact location of the laser spot measurement.

It may be useful at this point to directly compare the two techniques used during the study as summarised in Table 3. The advantages and disadvantages given concern the specific equipment used in this study. Differences in equipment specification may alter the usefulness of a technique for example if HSI which produced reflectance spectra further into the IR was used instead then the identification of pigments could be done more independently of a secondary technique.

**Table 3:** A summary of differences between Raman spectroscopy and hyperspectral imaging as used in this study

	Raman spectroscopy	HSI
Analysed area	Spot size: 30 µm diameter	Pixel size: 13,000 µm across
Counting time by spot or surface unit	≈ 5 min	≈ 0.33 mm s^−1^
Irradiance	0.4 mW	≈ 3.5 lux h
Advantages	Identification can be made unequivocally through the use of characteristic peaks	Identification relies upon the comparison of reflectance spectra and must be backed up by another method of analysis
Disadvantages	Limited spots can be analysed in a reasonable time frame	Larger areas can be scanned in a reasonable time allowing the user to ‘map’ important features

## Results

Table 4 shows the distribution of the Raman spectroscopy analysis across the six manuscripts and the pigments which were identified using Raman spectroscopy. These results are taken as correct and were compared to the results generated by the characterisation of reflectance spectra from hyperspectral data cubes by way of the three computational algorithms designed to compare experimental spectra to those in a database 5. Such databases were used as detailed above in “HSI database” section. If the algorithm identified the same pigment as Raman spectroscopy then it was considered correct, in this way a percentage success was derived for each configuration, as detailed in “Workflow” section.

**Table 4:** The results of the Raman spectroscopy survey showing the distribution of inspection points across the manuscripts involved in the study

Shelf mark	Pigments identified through Raman spectroscopy						Total
	Red lead	Red lead + vermillion	Lapis	Indigo + orpiment	Vermillion	indigo	
MS Arm g4	1	1	3	0	2	0	7
MS Arm e34	1	0	5	0	2	0	8
MS Arm d3	0	0	0	0	0	5	5
MS Arm d22	0	1	0	0	1	2	4
MS Arm c3	0	0	2	0	3	2	7
MS Arm d13	1	0	0	1	56	1	59
Total	3	2	10	1	64	10	90

Not all manuscripts had the same number of illuminations (MS Arm d13 was particularly lavishly decorated). Note the chemical formulas and common names; red lead (Pb_3_O_4_, minium), vermillion (HgS, cinnabar), lapis lazuli (S_3_, ultramarine (differentiation between synthetic (ultramarine), and natural (lapis) not possible with this Raman setup), orpiment (As_2_S_3_), indigo (C_16_H_10_N_2_O_2_)

The results of the Raman spectroscopy survey showing the distribution of inspection points across the manuscripts involved in the study. Not all manuscripts had the same number of illuminations (MS Arm d13 was particularly lavishly decorated). Note the chemical formulas and common names; red lead (Pb_3_O_4_, minium), vermillion (HgS, cinnabar), lapis lazuli (S_3_, ultramarine (differentiation between synthetic (ultramarine), and natural (lapis) not possible with this Raman setup), orpiment (As_2_S_3_), indigo (C_16_H_10_N_2_O_2_).

Table 5 shows the percentage success of the three algorithms with the five databases applied to all the manuscripts. The settings and parameters of the algorithms could be manipulated to increase the likelihood of a match by way of eliminating noise or focussing on characteristics of the spectra, and this was also done. Table 6 gives the results of the analysis carried out using default settings broken down into the different manuscripts.

**Table 5:** A comparison of the percentage agreement obtained using the different methods, databases, and wavelength ranges

Percentage success																
Database	1			2			3			4			5			Sample size
Algorithm	SFF	SAM	BE	SFF	SAM	BE	SFF	SAM	BE	SFF	SAM	BE	SFF	SAM	BE	
Default settings	20	9	13	71	49	37	60	23	22	27	14	18	43	44	44	91 (73 for databases 2 and 3)
Tailored wavelength range	66	47	67	89	89	93	89	81	92	81	66	65	89	87	84	91 (73 for databases 2 and 3)
400–800 nm	22	15	16	71	49	37	42	33	19	26	16	30	47	43	48	91 (73 for databases 2 and 3)
Difference (400–800 and default)	2	7	3	0	0	0	− 18	10	− 3	− 1	2	12	4	− 1	4	91 (73 for databases 2 and 3)
Difference (tailored and default)	46	38	54	18	40	56	29	58	70	54	52	47	46	43	40	91 (73 for databases 2 and 3)
Difference (tailored and 400–800)	44	32	51	18	40	56	47	48	73	55	49	35	42	44	35	91 (73 for databases 2 and 3)

The difference between 400–800 and default was calculated as the percentage success of the 400–800 nm range minus the percentage success of the default wavelength range, similarly Difference (tailored and default) was %tailored − %default and Difference (tailored and 400–800) was %tailored − %400–800

**Table 6:** Showing the percentage success of the databases and algorithms with the default wavelength range used separated out into the different manuscripts’ results

Percentage success	Default settings															
Database	1			2			3			4			5			Sample size
Algorithm	SFF	SAM	BE	SFF	SAM	BE	SFF	SAM	BE	SFF	SAM	BE	SFF	SAM	BE	
Total	20	9	13	71	49	37	60	23	22	27	14	18	43	44	44	91 (73 for databases 2 and 3)
MS Arm e34	38	25	13	N/a	N/a	N/a	N/a	N/a	N/a	38	13	0	38	38	25	8
MS Arm d13	10	7	9	68	45	34	55	13	13	32	20	27	49	47	44	59 (56 for databases 2 and 3)
MS Arm d3	100	20	20	100	60	80	100	60	80	0	0	0	0	80	80	5
MS Arm g4	0	0	0	N/a	N/a	N/a	N/a	N/a	N/a	0	0	0	43	29	14	7
MS Arm d22	50	0	50	50	50	0	50	50	25	0	0	0	25	25	50	4
MS Arm c3	29	14	43	100	86	57	86	71	57	43	0	0	43	29	71	7

The difference in sample sizes is due to databases 2 and 3 not being used on some folios from MS Arm D13, MS Arm e34, and MS Arm g4, this is discussed in the text

A comparison of the percentage agreement obtained using the different methods, databases, and wavelength ranges. The difference between 400 and 800 and default was calculated as the percentage success of the 400–800 nm range minus the percentage success of the default wavelength range, similarly difference (tailored and default) was %tailored − %default and difference (tailored and 400–800) was %tailored − %400–800.

As can be seen the majority of pigment regions identified were vermillion: this is because Raman spectroscopy very easily identifies this pigment but also because the Armenian manuscripts analysed here are very red in colour, so vermillion is probably the most prolific pigment throughout the manuscripts. It can also be seen from Table 5 that the majority of pigments were taken from MS Arm d13. This is because there were so many illuminations in this manuscript compared to the others, thus it provided a wealth of experimental material.

For example focussing on results from Database 1 (Table 5) we can see that using the algorithms on a “tailored” wavelength range increased the accuracy of reflectance spectra identification by 38–54%. The best algorithm in this case was the most simple algorithm, binary encoding (67% success) when used over a tailored wavelength range. SAM was the worst algorithm if applied with the default settings (9% success). This is true, on the whole, for all the databases with the best percentage success being for Database 2, BE, Tailored wavelengths (93% success), and the worst being 9% as above. Spectral feature fitting (SFF) scored higher than spectral angle mapping (SAM) in most cases and is also the one most likely to be correct under default settings which suggests that SFF deserves more consideration when analysing hyperspectral data, presently SAM is the more utilised algorithm.

Database 2 was provided the most matches under all parameters: we can also see that Database 5 was provided more than Database 4. These results lead us to believe that the database most likely to produce a correct characterisation is one which contains spectra obtained from material matching the experimental material as closely as possible (same binder, same age paper, same age pigments etc.) and containing only the most relevant spectra i.e. a database can be too large for the application.

Table 6 shows us the results for all three algorithms applied to all six manuscripts, but only for the default wavelength range. Here we see as before that on the whole BE was the least successful whilst used across the full wavelength range available. This trend is expected because binary encoding would only really work on data in which the peak is the main feature. In this data that is not the case however as noted above it does improve when the algorithm is focussed on a smaller wavelength range. This too is expected from BE because the main absorption feature would be focussed upon becoming a larger factor in the algorithms’ final result. As before we see that SFF is commonly the most successful for the full wavelength range. It has achieved 100% in places but it is to be noted that these datasets are rather small on their own. On the largest dataset, that of MS Arm d13, SFF still outperformed the others, but was far from achieving perfection. It is interesting then that SAM has become the more popular algorithm.

Showing the percentage success of the databases and algorithms with the default wavelength range used separated out into the different manuscripts’ results. The difference in sample sizes is due to databases 2 and 3 not being used on some folios from MS Arm D13, MS Arm e34, and MS Arm g4, this is discussed in the text.

It is interesting to note from Table 6 that seemingly the most difficult manuscript to obtain high percentage results from is MS Arm g4, one of the later manuscripts. It should be remembered though that the best databases (as before Databases 2, and 3 outperformed the others) could not be used on the ROIs from this manuscript because it was from these ROIs that the databases were formed, and this perhaps has created challenges. It would be interesting to obtain more ROIs for MS Arm g4, and MS Arm e34 for application of Databases 2, and 3 to these manuscripts. No manuscript consistently provided a greater percentage accuracy than others.

## Discussion and conclusions

In this paper, we have assessed the ability of HSI to identify pigments based on their reflectance spectra (380–1000 nm). Results were compared directly with Raman spectroscopy and for the first time an attempt was made to provide a percentage accuracy for this use of HSI in this application. This percentage accuracy was found to vary between 9 and 93% in total dependent upon the configuration of the algorithms applied to the data and the database used for characterisation. The best use of the algorithms required a great deal of manipulation of algorithm parameters, thus lending itself not to identification, but to mapping known pigments. The database most likely to provide a high percentage accuracy was one that was as close as possible to the pigments studied, and contained only relevant spectra. Databases 2, and 3 were consistently high performers, interestingly their data was taken from the most recent manuscripts.

This data highlights some pigment knowledge is needed to be certain that HSI is correctly characterising data, and that best practice would be to use techniques such as Raman to identify pigments, and HSI to map the pigments across the surface of the manuscript, or conversely for the mapping of areas of interest prior to spot analysis techniques. Therefore, based on this research, HSI should not be used exclusively to give an overview of pigments but should be used in conjunction with other techniques.

**Fig. 6:**
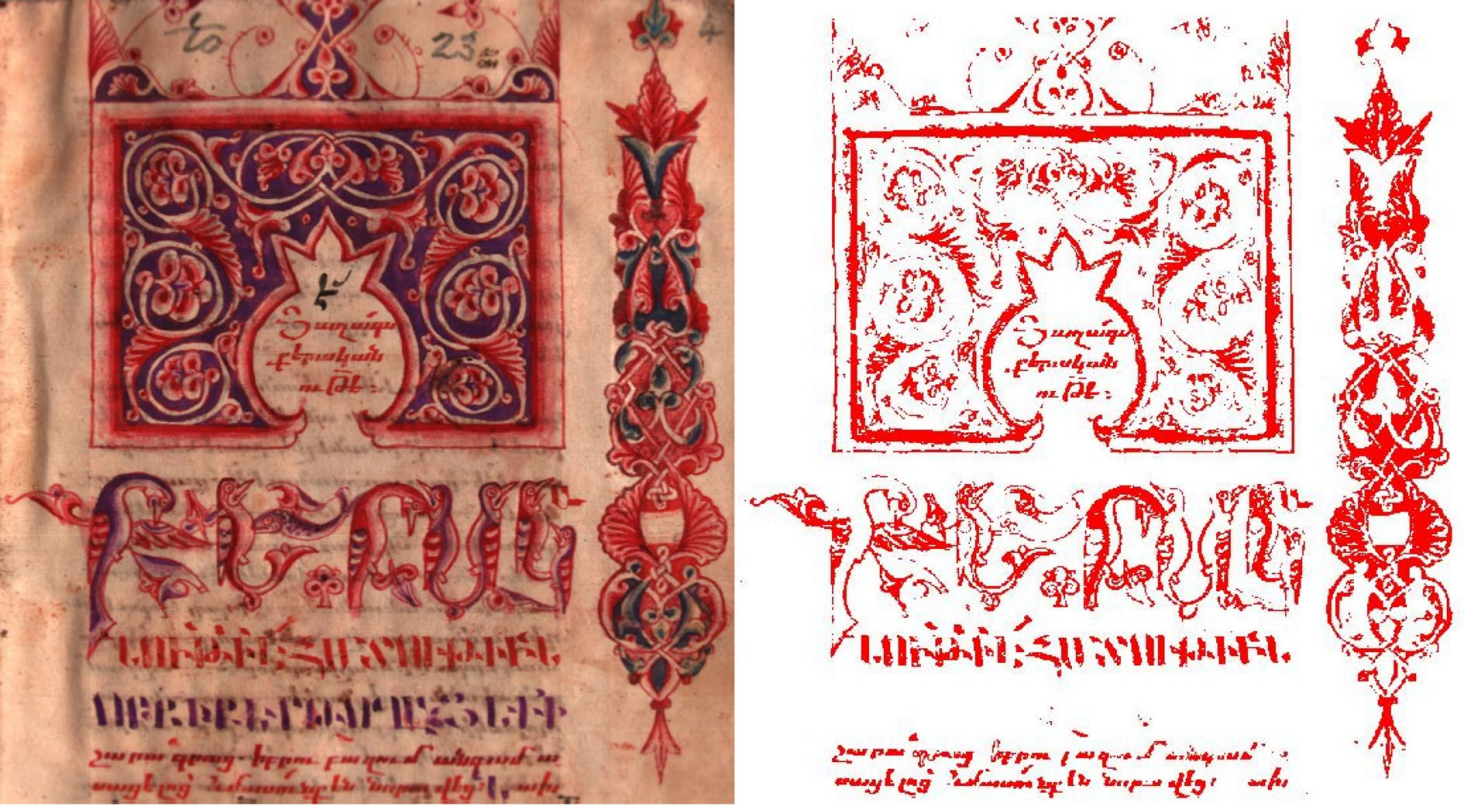
Showing the map of vermillion pigment across MS Arm e34, folio 4r using SAM

Other studies have used HSI successfully to identify pigments but they have always applied another technique to aid the identification, and occasionally a larger wavelength detection range has been used, detecting longer IR wavelengths [6, 14]. This study confirms that the visible wavelength range is not enough for a characterisation but it does offer hope that using hyperspectral data to map pigments can be accurate if used in conjunction with other techniques, which is our major recommendation. One such pigment map is given below in Fig. 6.

From this research HSI appears to not be as accurate for pigment analysis as Raman spectroscopy on a point by point basis but on the other hand HSI is vastly quicker at scanning a large area: we therefore recommend the use of the two processes together if a full quantification of the surface is required, with Raman for identification of pigments, and HSI to map, confirm similarity over a large area, or to identify areas for point analysis as has been done in the past [39, 40]. For HSI the accuracy of pigment identification could be improved by increasing the range of wavelengths scanned and by way of a more relevant database, again this would require the use of additional techniques such as Raman spectroscopy, suggesting that the best results are obtained firstly by making sure that the techniques at hand are used for purposes suiting their limitations, and secondly that a combination of techniques will yield superior results in a more efficient timescale. In general hyperspectral imaging could also benefit from greater spatial resolution and increased ease of use if it were to be used more frequently in a heritage environment.

A possibility for extending the study further would be to investigate and compare the accuracy and efficiency of other techniques (FTIR for example) and more work could be done with the SFF algorithm which gave a higher percentage accuracy here than the more popular SAM algorithm. This was expected for the reflectance data produced. SFF is designed to work best on absorption features such as those seen. It is perhaps surprising that SAM performed so poorly given its popularity but this could be explained by a lack of spectral features in general and the fact that the default settings in this software would be geared towards remote sensing data. Binary encoding did better than SAM, and this is perhaps because the simplistic algorithm did not require much adjustment.

Only one wavelength was used for the excitation laser for Raman spectroscopy, 635 nm. Previous studies [50, 85, 86] have shown that different lasers may increase the identifiable range of pigments, with 532 and 785 nm being other commonly used wavelengths. 785 nm has proven to be the most effective at pigment identification but requires an increase in the applied power [50, 85, 86] (to achieve good S/N ratios) which can cause damage to the object of analysis [50]. 532 nm has been shown to be better than 635 nm only for the identification of blue pigments [50, 85] but in general suffers from increased fluorescence [86]. It is therefore possible that the use of an excitation laser with a wavelength of 785 nm would identify more pigments, but it may also require the application of more power than we are comfortable with for valuable historic documents. 532 nm would be the logical one to try but could be of limited use because it is not as good all round and the manuscripts are predominantly red. The study compares HSI to Raman, if more pigments were found with Raman then more comparisons could be made, this would obviously have an effect on the end result, though it is difficult to predict how. In a similar vein a study incorporating manuscripts from of a more varied origin would expand the study in terms of pigments analysed (Armenian artists used lapis lazuli more prolifically than others for example) but on the whole the study results reflect the dynamic between two techniques and is applicable to other investigations, especially considering similar situations have been shown in the literature.

HSI has a great potential to be useful in the analysis of pigments. The database used was shown to be the most important single factor in increasing the match and a larger quantity of spectra but a smaller more focused number of pigments i.e. ones relevant to the object of study in terms of its chemical composition gives the best results. Caution must be used however, and a combination of analytical techniques is required to properly characterise a pigment and 93% was only possible with prior knowledge of the target pigment. Using default settings, the percentage accuracy was not sufficient.

When studying such documents HSI is a great advantage, it is a non-destructive technique which is capable of efficiently mapping the entire surface of an object. When a combination of techniques is used the setup can be a very powerful investigative tool and we recommend using HSI as a mapping tool prior to other techniques, or after them to give a complete picture of the pigment distribution. On a point by point basis however, point techniques such as RS offer clear advantages.

## Additional file

**Additional file 1.** Additional Figures S1–S5 and Tables S1, S2.

## Abbreviations

HSI: hyperspectral imaging
RS: Raman spectroscopy
FORS: fibre optic reflectance spectroscopy
FT-IR: Fourier transform infrared spectroscopy
HPLC: high performance liquid chromatography
GCMS: gas chromatography mass spectrometry
SAM: spectral angle mapping
BE: binary encoding
SFF: spectral feature fitting
ENVI: Environment for visualising images
XRF: X-ray fluorescence spectroscopy
ROI: region of interest
UV–VIS: ultraviolet–visible light

## Footnotes

1 A class of organic pigments made by precipitating a dye with a binder, the name derives from lac (a resinous secretion) and so is an interesting misnomer, having nothing to do with bodies of water.

2 The Mulholland et al. study used the same hyperspectral equipment as ours, but there are key differences between the studies. Firstly Mulholland et al. used additional NDT methods namely XRF, and FORS. Secondly Mulholland et al. focussed on the use of hyperspectral data to generate false colour composites whereas our study focusses on the characterisation of the reflectance spectra generated thus the two studies are useful companion pieces each demonstrating different functionalities of the data.

3 Pigments explained: Gold: Usually gold leaf but also shell gold which is powdered gold mixed with a binder, so named because it was traditionally prepared in a shell. White lead: The oldest white pigment, a by-product of lead production. Vermillion: A red man-made pigment of mercuric sulfide. Orpiment: A yellow pigment made from heating and grinding the orpiment stone. Ultramarine: Significantly also called lapis lazuli, made from a semiprecious stone of the same name. Red Lake: A transparent red made from the root of the Madder plant (Rubia Tinctoria) hence it is also called madder. http://www.jcsparks.com/painted/pigment-chem.html#Lapis.

4 ENVI: Environment for visualising images (accessed via University of Oxford site license), is a software package for remote sensing and dealing with hyperspectral datacubes containing a number of statistical and image processing algorithms. ENVI is a good choice for this investigation. It does not require the operator to code the algorithms prior to use and yet adjustments to the algorithms (in this case SAM, SFF, and BE) can be made to suit the user. This ease of use helps to simulate a situation where a non-expert might use the equipment, as is likely the case. Further work could have included the use of additional algorithms (KM theory for example) but the algorithms used in this study, particularly SAM, are commonly used (as has been shown in the literature) and there would be little advantage to writing our own SAM algorithm when one was readily available to us.

5 Statistical analysis methods: Briefly, SFF, or spectral feature fitting, is a least squares technique which uses methodology based on the absorption features of the spectra [67]. Spectral angle mapping, or SAM, treats each spectrum as a vector and calculates the angle between two spectra, thus determining spectral similarity. Smaller angles represent better matches and the technique is relatively insensitive to illumination effects [68, 69]. Binary encoding (BE) samples the data based on whether a band falls below or above the spectrum mean: the greater the number of bands which match the reference spectrum, the more likely it is that the spectra match [70].

6 Not all data was collected at first, and not all databases were created initially, below is a summary of the timeline of the experiment showing in what order, and why things were done throughout the experiment. The databases are more fully described in “HSI database” section. 1. An initial study using Raman spectroscopy (34 points from across the six manuscripts), carried out by Andrew Beeby (see “Acknowledgements” section), was performed for the purpose of including results in the exhibition “Armenia: Masterpieces from an enduring culture” and for the purpose of learning more about an infrequently studied set of manuscripts. 2. The data gathered was used to trial the methodology outlined in “Workflow” section: a. Hyperspectral scans were taken of MS Arm g4, MS Arm e34, and MS Arm d13 b. Reflectance spectra database 1 was created to carry out the identification of the hyperspectral scans (g4, e34, d13). c. Greater agreement between the two techniques was desirable and so it was thought that if an improvement were made to the database so the pigment library contained spectra closer to the ones taken from Armenian manuscripts. 3. Databases 2, and 3 were created using reflectance spectra from the ROIs in the hyperspectral scans of MS Arm g4, e34, d13 and therefore could not be used to test these ROIs again as the spectra is an exact match which would cause bias in the results. 4. Databases 1, 2, and 3 were tested upon hyperspectral data from MS Arm g4, e34, d13, d3, d22, and c3 with the exceptions of the use of databases 2, and 3 on the ROIs mentioned above. 5. Additionally the computational comparison algorithms for reflectance spectroscopy were configured using different parameters in order to investigate the effect of these changing parameters on the results with a view to increasing the frequency with which the hyperspectral identification pipeline agreed with Raman spectroscopy. 6. Additional Raman spectroscopy data was collected (56 additional points) in order to expand the corpus. 7. Additional hyperspectral imaging scans were taken to complement the additional Raman spectroscopy data. 8. Databases 4, and 5 were created using reflectance spectra downloaded from an online database (http://e-conservation.org/issue-2/36-FORS-spectral-database [65]). Database 4 contains all the spectra in the database, database 5 contains only those most relevant to the Armenian manuscripts which was done to demonstrate the difference that a change in database such as this can cause to the results obtained from the reflectance spectra comparison algorithms. In both cases the “egg tempera” database was downloaded for use as this is the binder most likely used in the Armenian manuscripts studied.

7 http://www.kremer-pigmente.com/en/ Kremer Pigmente is a manufacturer and distributer of historic pigments.

8 No processing was done on the downloaded spectra however the computational analysis does carry out some pre-processing for example ENVI documentation reveals that when SFF is carried out the spectra are “scaled to match the image spectra” http://www.harrisgeospatial.com/docs/SpectralFeatureFitting.html.

9 Spectralon^®^ is the brand name of a fluoropolymer material which has a very high diffuse reflectance (99%) and reflects evenly across the UV–Vis-NIR range [72].
