# Supplementary material for: Comparing the effectiveness of hyperspectral imaging and Raman spectroscopy: a case study on Armenian manuscripts
Source: Herit Sci. 2018 Jul 6;6(1):42. doi: 10.1186/s40494-018-0206-1 (PMC6559133; doi:10.1186/s40494-018-0206-1)
Supplement: Supplementary file 1 — Additional file 1. Additional Figures S1–S5 and Tables S1, S2. [file 40494_2018_206_MOESM1_ESM.docx]

Additional file 1

Methodological example

First Raman Spectroscopy is used to identify a pigment: Shown below in Figure 1 is the USB microscope capture of region of interest (ROI) 1, MS Arm d13, folio 33 and the Raman spectroscopy spectrum obtained from that spot.

Figure 1. a) The image taken with the USB microscope prior to Raman spectroscopy being performed on the spot. The laser spot from the Raman spectroscopy can clearly be seen pointing at a red area of the border b) The Raman spectrum obtained from analysis at that point is shown in red, beneath it in black are the components of the peak separated for clarity. The peaks are characteristic of Vermillion and the broad feature is caused by fluorescence, these combine to give the spectrum shown. The region of interest is in the top right of the folio, the full folio is shown in Figure 2 below.

Secondly HSI is performed on that folio, the hyperspectral scan for this example is given below in Figure 2.

Figure 2. An RGB composite (R: 639.9560 nm, G: 549.8530 nm, B: 469.9740 nm) of the HSI scan of MS Arm d13 folio 33. Note the colours appear to be slightly different to those shown in the Raman spectroscopy’s accompanying USB microscope image. This is because the image is a 3 colour composite generated by selecting 3 wavelengths from the 972 acquired from HSI and applying false colour.

Next using ENVI, a region of interest is created by selecting pixels which the user wishes to examine. These pixels are selected to exactly correspond to the area examined by the Raman spectroscopy laser, as shown by the USB microscopy photo (Figure 3).

Figure 3. A region of interest (ROI) is generated in ENVI by selecting desired pixels, compare this to the area shown to be under examination by Raman spectroscopy in Figure 2 above.

ENVI is then used to generate a reflectance spectrum for the ROI, by averaging the spectra of the individual pixels (Figure 4).

Figure 4. Showing the reflectance spectrum for ROI 1, MS Arm d13, folio 33. At this point the spectrum has not been classified but from the spectrum it is clear that it would appear red to the eye, as the blue and green wavelengths are clearly absorbed.

Finally this reflectance spectrum is compared, using the three algorithms discussed, to the locally stored HSI database of reflectance spectra. The software provides a list of spectra which it considers to be matches, with the most likely at the top, and the least likely at the bottom, each match is given an arbitrary number from 0 - 1, which is the result of the computational process. The higher this number, the more closely the reflectance spectrum analysed matches that spectrum in the database (Figure 5). Occasionally the database would contain multiple spectra of the same pigment, in this instance the software performs the same as it would if there were only one spectrum for each pigment choosing the closest match to the experimental spectrum, Figure 5 is an example of this situation. Of course if all the vermillion spectra in the database are at the top of the list one can be more certain of the identification than if the list given by the software contained vermillion spectra interspersed with spectra from other pigments in the list i.e. if there were no clear spectra match which was significantly more likely. This exemplifies list’s function. It can only suggest the most likely pigment from the ones about which it knows, it does not give a definitive answer, indeed different experimental spectra from areas identified as vermillion will produce different results in the list, this is because of the variability of reflectance spectra and demonstrates the need for the use of complementary techniques.

Figure 5. a) the most closely matched vermillion spectrum from the database (database 2 in this case) is compared to the spectrum of the ROI (in this case using the SFF algorithm). b) you can see from the results of the algorithm that all the likely matches are Vermillion in this case. A positive result.

This is carried out once for every database (of which there are 5), and every computational algorithm (of which there are 3), on each spot analysed by Raman spectroscopy. 90 spots were analysed for databases 1, 4 and 5. For databases 2 and 3 only 72 spots were analysed (because the other 17 ROI spectra were used for the creation of databases 2 and 3). These procedures were then repeated twice, using different boundaries for the wavelengths analysed by the computational algorithms (see below). In total this means that 3726 “database queries” were run.

The results of the database queries for reflectance spectra are then directly compared to the results from Raman spectroscopy as per section 3, above. A small sample of this data is reproduced below in Table 1.

**Table 1.**

A sample of the final results table. This section shows the Raman and the Hyperspectral results for the regions of interest in MS Arm g4 using database 1 for the hyperspectral (reflectance) data. The reflectance results which agree with Raman spectroscopy are highlighted in green. The default settings for this dataset are unsatisfactory, providing no correct results. The final results table contained 3726 database queries, this is just 81 database queries (2% of the total).

**Table 2**

| **Spectra** | **Score (SFF)** |
| --- | --- |
| Vermillion | 0.746 |
| Vermillion | 0.707 |
| Vermillion | 0.481 |
| Vermillion | 0.337 |
| Red Lead | 0.141 |

**SFF comparison ranking.** From this the analyst sees that the most likely candidate for the identity of the pigment is vermillion (according to spectral feature fitting) and that it is unlikely to be red lead. This table is generated by comparing the experimental reflectance spectrum to the reflectance spectra stored locally in the hyperspectral reflectance spectra database. This database contains multiple spectra identified as vermillion which is why there are multiple comparisons to vermillion in the table. As you can see some vermillion spectra in the database are a closer match to the experimental spectra than others in this case. The experimental spectrum is unlikely to be red lead.
